# The Regulatory Properties of the Ccr4–Not Complex

**DOI:** 10.3390/cells9112379

**Published:** 2020-10-29

**Authors:** Nafiseh Chalabi Hagkarim, Roger J. Grand

**Affiliations:** Institute for Cancer and Genomic Sciences, The Medical School, University of Birmingham, Birmingham B15 2TT, UK; N.ChalabiHagkarim@bham.ac.uk

**Keywords:** Ccr4–Not complex, mRNA decay, deadenylation, transcription, cell cycle, chromatin modification, RNA export, DNA damage repair

## Abstract

The mammalian Ccr4–Not complex, carbon catabolite repression 4 (Ccr4)-negative on TATA-less (Not), is a large, highly conserved, multifunctional assembly of proteins that acts at different cellular levels to regulate gene expression. In the nucleus, it is involved in the regulation of the cell cycle, chromatin modification, activation and inhibition of transcription initiation, control of transcription elongation, RNA export, nuclear RNA surveillance, and DNA damage repair. In the cytoplasm, the Ccr4–Not complex plays a central role in mRNA decay and affects protein quality control. Most of our original knowledge of the Ccr4–Not complex is derived, primarily, from studies in yeast. More recent studies have shown that the mammalian complex has a comparable structure and similar properties. In this review, we summarize the evidence for the multiple roles of both the yeast and mammalian Ccr4–Not complexes, highlighting their similarities.

## 1. Introduction

The yeast Ccr4–Not complex is a large (1.9-MDa) and highly conserved multifunctional assembly of proteins, involved in different aspects of mRNA metabolism. These include the repression and activation of transcription initiation, control of mRNA elongation, deadenylation-dependent mRNA turnover, and in ubiquitin-protein transferase activity reviewed [1,2,3,4,5]. The activation of the complex can be seen in various ‘downstream effects’ such as histone methylation. Most of the original studies concentrated on the role of the Ccr4–Not complex in *Saccharomyces cerevisiae*. In yeast the complex has nine core subunits, comprising Ccr4 (carbon catabolite repression), Caf proteins (Ccr4 associated factor) (Caf1, Caf40, Caf130) and Not proteins (Not1, Not2, Not3, Not4, and Not5) as well as several less strongly associated components which are probably interacting partners [6,7] (Table 1). Not1 is the largest subunit of the complex (>200 kD) and forms a scaffold for the other components [8]. No clear function has been assigned to the Not module comprising Not2, Not3 and Not5 although it does act as a cofactor for the deadenylation activity and may be involved in interaction with the ribosome [9,10]. Genetic approaches in yeast have also demonstrated that the Not1–4 genes can globally repress RNA polymerase II activity. The mutation of these genes increases the basal expression of many genes [11]. The Ccr4-Caf1 mRNA deadenylase complex contains a 3′ exoribonuclease which is involved in removing poly (A) tails from mRNA [12,13,14,15,16]. Not4 is responsible for the second major enzymatic activity of the complex, E3 ligase-mediated ubiquitylation [17].

Complexes of a comparable size, containing the human orthologues CNOT1–CNOT9 with three additional subunits of CNOT10, Tab182 (Tankyrase 1-binding protein1, TNKS1BP1) and C2ORF29 (CNOT11), have been identified in mammals (Table 1). Four deadenylase subunits are expressed in mammalian cells. These appear to form various heterodimers, CNOT7/CNOT6, CNOT7/CNOT6L, CNOT8/CNOT6, and CNOT8/CNOT6L. Thus, the complex contains either CNOT7 or CNOT8, suggesting they compete for binding to CNOT1 [18,19]. While the E3 ubiquitin ligase Not4 is consistently present in the yeast complex, CNOT4 is not as stably associated as the other subunits in mammalian cells [18]. No orthologues of CNOT10, CNOT11, or Tab182 have been identified in yeast [20]. The CNOT3 subunit, with no specific enzymatic activity, is orthologous to two yeast subunits, Not3 and Not5. Every individual subunit appears to have a unique role with a slight overlap between some proteins [21]. The evidence in support of this includes the observation that mutations and deletions of each different subunit are responsible for different phenotypes in yeast [21].

Throughout this review, we have focused, primarily, on the biological roles of the complex in a variety of contexts. However, it should be noted that some effects can be ‘direct’, caused by the Ccr4–Not proteins themselves, either singly or as a component of the complex, or ‘indirect’ as a result of changes induced in target pathways. For the sake of completeness, we have considered the structure of the complex but a detailed structural description has not been presented here.

## 2. Structure of the Ccr4–Not (CNOT) Complex

As with any large multi-subunit assembly of proteins considerable effort has been expended to determine the structure of Ccr4–Not. Up to the present, detailed structural information is available for the complex from four species: *Schizosaccharomyces pombe*, *Saccharomyces cerevisiae*, *Drosophila melanogaster*, and *Homo sapiens.* Limited data are also available for the thermophilic fungus *Chaetomium thermophilum* complex [9,14,16,22,23,24,25,26,27]. In all cases, it has been shown that CNOT1, the largest subunit (>200 kD molecular mass), forms a scaffold for the complex and most, although not all, other components bind to it directly [28]. However, a number of obvious differences between the species have been identified. Firstly, there are four deadenylase components in the human complex (CNOTs 6, 6L, 7 and 8) but only two in yeast (Ccr4 and Caf1). The Ccr4-Caf1 mRNA deadenylase complex contains a 3′ exoribonuclease motif [12,13,14,15,16]. Secondly, it appears that the E3 ligase protein, CNOT4/Not4, is quite strongly associated with the complex in yeast but not in *Drosophila* or humans [17,18,29]. Thirdly, additional components have been identified in the *Drosophila* and human complexes. These are CNOT10 and CNOT11 (C2orf29). TNKS1BP1 (Tab182) also seems to be a member of the human complex [18,20,30] (Table 1).

The complex, of approximately 1.9 M molecular weight in yeast, is L-shaped with two arms of approximately equal length (180–190 Å) and a hinged region present in the centre [22,24].

**Figure 1:**
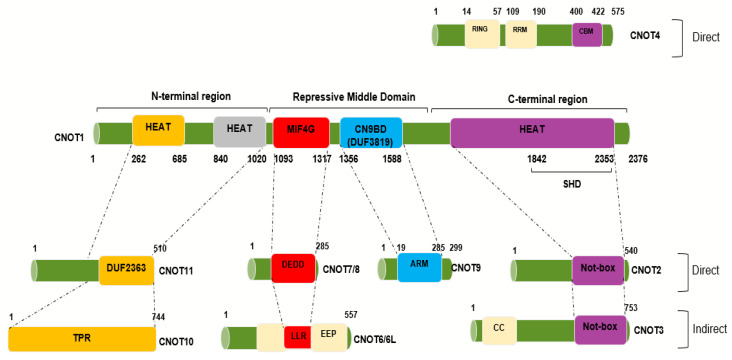
Human CNOT1 interaction regions with the other CNOT subunits. Interaction map obtained from negative-stain electron microscopy showing the interaction sites between CNOT1, acting as a scaffolding platform, and the other CNOT subunits. The human homologue, CNOT1, encompasses 2376 amino acids and is 20% identical (32% similar) to its yeast counterpart. Different colors represent the different regions as shown above. The human Tab182 interacting site has not been mapped yet. Negative on TATA-less (NOT), Middle domain of eukaryotic initiation factor 4G (MIF4G), Domain of uncharacterized function (DUF3819 and DUF2363), Exonuclease-endonuclease-phosphatase (EEP)-DNase I-like domains, Death effector domain-containing protein (DEDD)-RNase D-like domains, Leucine-rich region (LRR)-RNase D-like domains, Superfamily Homology Domain (SHD), Armadillo (ARM) Repeat Domain, predicted coiled-coil domain (CC), Predicted domain consisting of α-helical TPR-like repeats (TPR), CAF40-binding motif (CBM) (Adapted from [25,31,33,34]).

The complex is assembled on the NOT1 backbone. How the other components associate has been determined using in vitro protein binding studies, crystallography, and electron microscopy. Binding sites for the other components of the complex on CNOT1 appear to consist of α-helical domains. A number of structural sub-complexes have been delineated-these comprise the deadenylase module (Ccr4 and Caf1 in yeast, CNOT6/CNOT6L and CNOT7/CNOT8 in humans), the NOT4 (Not4) the E3 ligase module and the ‘Not module’ (Not2 and Not5 in yeast, CNOT2 and CNOT3 in humans). The binding sites of additional subunits have also been mapped-thus, metazoan CNOT10 and CNOT11 bind to the N-terminal domain of CNOT1 and CNOT9 (Caf40 in yeast) binds to the central region of CNOT1 [16,20,25,31] (Figure 1). The C-terminal region of CNOT1 forms a rigid structure, which comprises two perpendicular stacks of HEAT-like repeats. CNOT2 and CNOT3 each contain SH3-like Not box domains which provide dimerization sites. CNOT2/CNOT3 binds to the CNOT1 C-terminal region [23,28] (Figure 1). The C-terminal binding site of CNOT1 comprises 10 HEAT repeats which contain helix A-turn-helix B motifs. The structures of the C-terminal complexes have been determined for the yeast and human proteins. CNOT2/Not2 and CNOT3/Not5 form a heterodimer through their Not-box motifs. A region of CNOT3/Not5 interacts with HEAT repeats 1–5 through hydrophobic and polar amino acids and a region of CNOT2/Not2 is spread across Not1 from HEAT repeats 9 and 10 and binding to repeats 4–6 [9,23]. Although no specific functions have been determined for this C-terminal module it is linked to the stability of the complex as a whole and the recruitment of mRNAs [9]. The C-terminal complex associates with synthetic ribonucleotides, such as poly(U) RNA, in vitro with a binding site comprising structural elements from Not1, Not2 and Not5 [9]. Boland et al. have also shown that an intact C-terminal module of the Drosophila CCR4–NOT complex is required for optimal mRNA degradation [23]. Furthermore, the CNOT2/CNOT3 heterodimer can stimulate the deadenylase activity of the complex [23]. Recent evidence indicates that yeast Not5 directly associates with the ribosome and, together with Not4, plays an important role in the regulation of mRNA half-life (Section 3) [10].

CNOT4 (Not4) is an evolutionarily conserved E3 ubiquitin ligase [17,29]. It contains a RING domain, a linker region which tends to form a coiled-coil, an RNA recognition motif (RRM) domain, and a C3H1-type zinc finger domain (ZNF) [31]. These motifs are all present within the conserved N-terminal region of human CNOT4. The C-terminal region of metazoan Not4 is variable in sequence but contains a conserved Caf binding motif (CBM) through which it binds directly to Caf40 (CNOT9) [31]. Flanking sequences to the CBM are involved in interaction with Not1 as well as assisting with Caf40 binding. This motif is not present in the yeast Not4 proteins although a Not1 binding site has been described [9]. It is not fully clear why yeast Not4 associates strongly with the full complex whereas the human and *Drosophila* proteins do not. However, it has been shown that the C-terminal region of human CNOT4 (Not4-C) is able to bind to the complex [31]. It has been suggested that the N-terminal region, therefore, somehow prevents Not4-C from interacting in the human complex. This could be explained by possible post-translational modifications or additional binding partners [31]. As well as binding to Caf40/CNOT9 the Not4-C also interacts with the C-terminal HEAT region of CNOT1, with a relatively slight contribution from CNOT2 and CNOT3 [9,31] (Figure 1). A number of substrates for ubiquitylation by CNOT4 have been identified and these are discussed elsewhere in this review (Section 4). Significantly, CNOT4 is required for optimal deadenylation activity by the full human CNOT complex, although a short C-terminal peptide will substitute for the whole protein [31].

The CNOT9 (Caf40) subunit binds to a central CNOT1 coiled coil domain (termed CN9BD or DUF3819). This is adjacent to the CNOT1 MIF-4G (middle portion of eIF4G) region (Figure 1). The human CNOT9 monomer contains six armadillo repeats forming a solvent-accessible, positively-charged cleft 21–22 Å wide [32]. Armadillo repeats are normally involved in protein-protein interactions but it has been shown that CNOT9 can also bind certain oligonucleotides in in vitro assays [32].

The deadenylase module associates with the central HEAT repeat-containing MIF-4G region of Not1. Thus, Caf1 (CNOT7) binds to Not1 through pre-existing structural motifs, made up of conserved hydrophobic residues. This allows full access for RNA molecules to the active site on Caf1 [15,16]. The leucine rich repeat (LRR) domain of Ccr4 (CNOT6L) binds to Caf1. Caf1 interacts with Ccr4 via a surface formed by a long loop and an α-helix. This region of Caf1 undergoes a localized conformational change compared to the unbound structure [16]. In humans, two additional deadenylase components are present in the complex, CNOT6 and CNOT8, and it seems likely that they bind to CNOT1 in a comparable way to CNOT6L and CNOT7 (Figure 1).

One of the obvious differences between the yeast and metazoan CNOT complexes is the presence of CNOT10 and CNOT11 (C2orf29) in the latter complex. Human and *Drosophila* CNOT11 binds to the N-terminal region of CNOT1 and CNOT10 binds to it [20]. The area of Not1 between the MIF-4G region and the N-terminal region comprises thirteen HEAT domains. The Not1 a.a.154–753 structure has been shown to contain antiparallel helices assembled side by side to form an elongated molecule. It has been suggested that these form structures ideal for protein–protein interactions within the complex or for binding of other molecules [16]. It should be noted, however, that this structure was determined for yeast CNOT1 which has no CNOT10 and 11 orthologs. Although CNOT10 and 11 do not appear to have any enzymatic activity of their own, their presence stimulates deadenylation through stabilization of the RNA substrate. It has been suggested that Caf40 (CNOT9) is the major enhancer of deadenylation, probably due to its proximity to the exonucleases, but if this is not available, CNOT10 and CNOT11 can compensate [25].

Depletion of CNOT1 results in the destabilization of the whole complex and degradation of some other subunits such as CNOT2, CNOT6L, CNOT7, and CNOT9, but not CNOT3, in HeLa cells [35]. Although, CNOT1 has no enzymatic activity, as far as is known, the importance of its scaffolding function for the deadenylase activities of the CNOT complex cannot be overstated. It has been shown that in CNOT1-depleted HeLa cells the level of CHOP mRNA increased and the cells undergo caspase-4 activation causing ER stress-mediated apoptosis, indicating CNOT1 is essential for viability and cell proliferation [35]. In addition, CNOT1 depletion in HeLa cells reduces the deadenylase activities and decreases the level of P-body formation, where mRNA decay is thought to take place [35].

What are considered to be the major constituents of the CNOT complex are described above. However, a number of other proteins have been routinely found to associate with the complex [6,7,36]. It appears that these components are not integral but are required for routine functions. For example, the BTG/Tob complex binds to CNOT7 and has a role in the recruitment of mRNAs [37]. Consequences of the interaction have been reported variously to be either activation or repression of deadenylase activity [38,39,40]. A large number of other RNA binding proteins (for example, Nanos2, Pumilio, Smaug and Tristetrapolin) act as adaptor proteins and interact with the CNOT complex and cause the suppression of target mRNAs [36]. Recent evidence suggests that Puf3 and Zfs1 associate with the complex and are critical in mRNA substrate selectivity as well as enhancing RNA binding [41]. Puf3 can distinguish between RNAs of very similar sequence and can therefore facilitate the ability of the Ccr4–Not complex to regulate the level of expression of particular target proteins [41]. Interaction of the complex with GW182 (a component of miRISC) through CNOT1 plays a role in miRNA-induced deadenylation [42,43,44]. The core subunit, CNOT1, has also been shown to associate with the translational repressor and decapping activator, the DEAD box protein DDX6 [34,45,46,47] (Section 3). DDX6 plays a role as a translational repressor in different pathways, including mRNA storage in erythropoiesis and microRNA mediated gene silencing [47]. Other proteins shown to interact with the CNOT complex include Bag-of-marbles, which represses the expression of specific mRNAs and binds to CAF40 (CNOT9), HIPK family kinases, which associate with the CCR4–NOT components CNOT2 and CNOT3 and phosphorylate the complex, the transcription factor EBF1, which binds CNOT3 and yTAFI, a core component of TFIID, which binds Not1 [48,49,50,51]. A number of these are discussed in more detail in the following sections.

Interestingly, it has been shown that the Ccr4–Not complex strongly associates with the YTH domain of nucleus-specific RNA binding subunit, Mmi1 (meiotic mRNA interceptor 1), close to the nuclease module, in *Schizosaccharomyces pombe* [24]. In fission yeast, Mmi1 is essential for viability. It represses the expression of transcripts and increases the deadenylation of target RNAs in in vitro assays [8].

## 3. mRNA Turnover and Deadenylation

Deadenylation-dependent mRNA decay plays an important role in eukaryotic fine-tuning of gene expression and this is achieved by monitoring mRNA translation and initiating degradation under appropriate circumstances [52,53,54]. Moreover, several studies have reported the role of deadenylation in the regulation of other cellular processes, including the DNA damage response (DDR) [55], cell cycle regulation [56] and cell proliferation [35]. The rate of deadenylation varies between different mRNAs and determines their half-lives.

Caf1 and its human orthologues CNOT7 (hCaf1a) and CNOT8 (hCaf1b) belong to the ribonuclease D (RNase D) group of the DEDD superfamily [4,57]. Amino acid sequence analysis of the CNOT7 and CNOT8 paralogues display a high degree of homology (76% identity and 89% similarity) [57,58]. While CNOT7 and CNOT8 can partially compensate for each other’s function, they also appear to have unique roles in other contexts [59]. For instance, CNOT7 null mice show defects in spermatogenesis, even in the presence of CNOT8, suggesting that CNOT7 and CNOT8 may not be entirely redundant in function [60,61]. In mammalian cells, the complex contains either CNOT7 or CNOT8, suggesting they compete for binding to CNOT1 [18,19]. 

The EEP-type nuclease family member Ccr4 and its mammalian orthologues CNOT6 and CNOT6L are also largely associated with deadenylase activity. Ccr4 is characterized by the presence of two highly conserved domains: a carboxy-terminal endonuclease-exonuclease-phosphatase (EEP) region and an amino-terminal leucine rich region (LRR) [57,62,63]. Yeast two-hybrid analysis initially demonstrated that CNOT6 directly binds CNOT7 and CNOT8, but not CNOT1. More recently, detailed structural analysis has confirmed this. The LRR domain is required for binding to Caf1 and integration into the CNOT complex [14,15,64]. Microarray analysis has shown that reduction of CNOT6 has little impact on mRNA levels compared to depletion of CNOT6L. For instance, depletion of CNOT6L leads to G1 cell cycle arrest following increased stability of the cell cycle inhibitor p27/Kip1. However, CNOT6 does not appear to be involved in regulation of cell proliferation [65] (see Section 8).

Deadenylase enzymes are characterized by their ability to promote rapid deadenylation in a 3′ → 5′ direction, resulting in the release of 5′-AMP. The yeast Ccr4–Not complex and the Pan2/Pan3 dimer function as two dominant deadenylase complexes, which are conserved in human cells [12,66,67]). The Pan2/Pan3 deadenylation complex initiates mRNA turnover by removing long poly (A) tails of above 150 nucleotides. A subsequent step is carried out by the Ccr4–Not complex which can remove the polyadenylate-binding protein PABPC1-bound A tails [68].

It has recently been shown that Caf1 and Ccr4 have distinct activities in yeast. Caf1 degrades naked poly (A) sequences but is blocked by PABPC/Pab1. However, Ccr4 is activated by PABPC to trim the PABPC/Pab1-protected regions [41,68]. The combined action of the two deadenylases delineates the 27 nucleotide periodic decrements seen along shortening tails. It has been concluded that Ccr4 is a general deadenylase that can trim all mRNAs whereas Caf1 is more specialized and deadenylates transcripts with reduced translation elongation and Pab1 binding [41]. Because of the stringency against non-A nucleotides Caf1 is unable to progress past the poly(A) tail [69]. The presence of, for example, guanosine inhibits deadenylation.

mRNA decay occurs within cytoplasmic ribonucleoprotein (RNP) granules known as processing bodies (P-bodies). Cytoplasmic mRNAs cycle between polysomes and P-bodies. P-bodies are distinct foci formed by phase separation within the cytoplasm and composed of translationally repressed mRNAs and enzymes related to mRNA turnover, including decapping enzymes (DCP1–DCP2 complex and NUDT16), ATP-dependent RNA helicase Dhh1 (yeast)Rck/p54 (DDX6) (mammalian), 5’-3’ exoribonuclease 1 (XRN1), the Lsm1–7 complex, Pan2/Pan3, the CNOT complex, along with the P-body component GW182 [70,71,72,73,74,75,76].

Formation of P-bodies is induced in response to stress and it has been shown that the localization of Ccr4–Not components to P-bodies is limited to stressed yeast and metazoan cells but not unstressed cells when decay is ongoing [77,78,79]. It has been hypothesized that the recruitment of Ccr4–Not components to P-bodies may trigger mRNAs for degradation and avoid extensively damaged mRNAs from re-entering the translatable pool until the stress is removed [80]. However, it has been shown that P-body formation is not essential for mRNA decay, NMD, and RNA-mediated gene silencing [79].

Evidence has been presented to show that components of the yeast Ccr4–Not complex play a role in decapping in P-bodies [81]. Human Ccr4 co-localizes in P-bodies with other degradation factors eIF4E-T, Lsm1, Rck/p54, but not Dcp2 [82]. In HeLa cells, the depletion of CNOT2 inhibits deadenylase activity of the CCR4–NOT complex and disrupts P-bodies although it is not a structural component; in male gonocytes CNOT3 can be recruited to P-bodies by Nanos2 [83,84]. 

ATPase activity of Dhh1 is required for the disassembly of P-bodies [85]. Not1 from its N-terminus interacts with the DEAD-box ATPase Dhh1 and stimulates its activity [85,86]. In budding yeast, mutations in Dhh1 that interrupt the association between Dhh1 and Not1 prevent P-bodies disassembly in vivo [85]. Similarly, DDX6, the mammalian homolog of Dhh1, can also be stimulated by CNOT1 [45].

Different types of mRNA decay can be distinguished: generic mRNA deadenylation, nonsense mediated mRNA decay (NMD), targeted mRNA decay and deadenylation-independent decapping. The Ccr4–Not complex is involved in all of these.

**Generic mRNA decay** occurs upon translation termination. It is not clear whether the CNOT complex is present during translation in an inactive form, and is then activated upon translation termination to promote shortening of the poly (A) tail, or is recruited upon translation termination to initiate deadenylation. However, one study has shown a series of events which couple mRNA decay to translation termination starting with the recruitment of translation termination eRF1-eRF3 factors and recruitment of deadenylase complexes Pan2/Pan3, and Ccr4–Not by the BTG/Tob family of proteins via their interaction with PABPC1 [41,52,68].

**Nonsense mediated decay (NMD)** is a translation-coupled mechanism that recognises and eliminates aberrant mRNAs, generally those containing a premature termination codon (PTC). Interaction between cap binding protein (CBP) and the Upf1 helicase activates the SMG1-mediated phosphorylation of Upf1 [87]. Phosphorylated Upf1 promotes recruitment of the SMG5/SMG7 heterodimer and SMG6 endonuclease to induce ribosome dissociation and decapping-mediated decay. The SMG5/SMG7 complex recruits the Ccr4–Not complex via an association between SMG7 and Pop2/CNOT8 (but not its homologue Caf1/CNOT7). The recruitment of Ccr4–Not by SMG7 is vital for the NMD surveillance pathway in SMG6-depleted cells, suggesting that SMG6 and the Ccr4–Not complex act redundantly to promote the degradation of NMD targets [88,89].

**Targeted mRNA decay** is the best-established function of the Ccr4–Not complex and this plays a key role in post-transcriptional gene silencing by its targeted recruitment to the 3′ untranslated regions (UTRs) of mRNAs. Genetic evidence shows that RNA-binding proteins (RBPs) and microRNAs have the ability to tether the Ccr4–Not complex to specific sequences at the 3′ end of the mRNA, thereby promoting poly (A) shortening and repressing translation and/or mRNA turnover [90]. 

CNOT1 mediates recruitment of the CNOT7/8 (Caf1/Pop2) and CNOT6/6L (Ccr4) deadenylases to the selected mRNA 3’UTRs through its interaction with specific RBPs thereby triggering degradation of target mRNAs [13,91]. For example, Tristetraprolin (TTP, zinc-finger protein ZFP36) controls the turnover of many inflammatory, hypoxia and oncogene signaling pathway mRNAs and functions as a tumour suppressor in lymphomas [92,93,94]. TTP directly interacts with CNOT1 and promotes the recruitment of CNOT7 to target mRNAs, such as those of tumour necrosis factor-α (TNF-α), Interleukin and transcription factors such as c-Fos and c-Myc, for degradation [91,95,96,97,98,99]. Similarly, CNOT6L functions as a negative regulator of human skeletal muscle differentiation by reduction in IL-8 mRNA stability [100,101]. High levels of IL-8 promote myogenic differentiation in rat skeletal muscle cells [36,102].

Several other examples of specific regulation of biological processes by Ccr4–Not-mediated deadenylation have been described. BTG/Tob adaptor proteins regulate mRNA decay through their interaction with CNOT7 and CNOT8 [56,103,104]. Indeed, post-transcriptional control of gene expression mediated by RBP-CNOT complexes is a key method of regulation during development. The RBP Nanos2 is implicated in maintenance and sexual development of germ cells [105,106]. Nanos2 directly interacts with the C-terminal domain of CNOT1 and plays a key role in murine male germ cell development by recruiting deadenylase subunits to meiosis-specific mRNAs, such as Stra8, for degradation in cytoplasmic P-bodies [84,107]. The significance of the Ccr4–Not complex is illustrated by the observation that depletion of CNOT1, CNOT2, or CNOT3 decreases mouse embryonic stem cell proliferation or viability [108]. In a genome-wide siRNA screen CNOT3 was identified among 148 genes whose expression is essential for self-renewal of mouse embryonic stem cells (ESCs) [109]. mRNA stability of cell death-related proteins, such as receptor-interacting protein kinase 1 (RIPK1) and RIPK3, is increased in CNOT3-depleted mouse embryonic fibroblasts (MEFs), leading to necroptosis [110].

The CNOT deadenylase complex is also involved in post-transcriptional control of gene expression during oogenesis and embryonic development in *Drosophila* [111,112]. *Drosophila* Ccr4 deadenylase acts with two translational repressors, Nanos and Pumilio, to promote germline stem cell (GSC) self-renewal by repressing the expression of differentiation factors such as meiotic P26 (mei-P26) [113]. mei-P26 is a major target of the Nanos/Pumilio/Ccr4 complex for GSC self-renewal, as GSC loss in twin (a homolog of the yeast Ccr4 deadenylase) mutants is rescued by reducing the gene expression of mei-P26. Twin regulates the length of cyclin A- poly (A) tail and that of bag-of-marbles (Bam), a regulator of cyst division, to permit *Drosophila melanogaster* oogenesis [114]. The stability of Nanos mRNA is also controlled by the CNOT complex through a mechanism in which the RBP Smaug binds to the Nanos 3′ UTR and recruits the CNOT complex, leading to rapid deadenylation and subsequent degradation of Nanos mRNA [115].

The miRNA repression complex, including GW182 (TNRC6) and Argonaute (AGO)-binding partners and effectors of miRISCs, has been shown to interact directly with CNOT1 as well as PAN3, leading to degradation of miRNA–target mRNAs [42,43,44,116,117]. CNOT1, through its MIF4G domain, binds to the C-terminal RecA domain of the DEAD-box of the RBP protein, DDX6, a central component of translational repression and decapping. It stimulates the DDX6 RNA helicase/ATPase and its recruitment to miRNA-targeted mRNAs [34,45].

Relevant to the role of Ccr4–Not in mRNA decay is the recent observation that the complex is physically associated with the ribosome in yeast [10]. Recruitment of Ccr4–Not occurs through Not5 which associates with the ribosomal E-site when the A-site is not occupied by tRNAs [10]. The interaction between the complex and ribosomes requires ubiquitylation of eS7 by Not4; the interaction occurs with both initiating and elongating ribosomes. Loss of Not5, eS25 or eS7 ubiquitylation results in dysregulation of mRNA half-life and impairment of decapping [10].

YTHDF2, the N^6^-methyladenosine (m^6^A)-binding protein, has been shown to associate with the SH domain of the CNOT1 subunit and triggers m^6^A RNAs for degradation [118,119,120].

It is important to remember when considering the effects of depletion or mutation of CNOT1, for example, that the deadenylase subunits are reliant on it for their activity. Thus, loss of the ability to reduce poly (A) tails may occur whilst the CNOT6, 6L, 7, and 8 components remain intact. Deadenlyation-independent decapping of certain mRNAs such as EDC1 mRNA, which encodes a protein that enhances decapping, is dependent on yeast Not2, Not4, and Not5 [81]. ATP-dependent RNA helicase and decapping activator Dhh1 directly interacts with N-terminus in Not1 and promotes the recruitment of Caf1 to target mRNAs, such as autophagy ATG mRNAs [86,121]. Thus, it could be that specific mRNAs require the CNOT complex to be efficiently decapped. Like Dhh1, Caf1 couples mRNA decay and translation by monitoring codon optimality [41,122].

## 4. The Ccr4–Not Complex and the Regulation of Protein Level: the Role of CNOT4/Not4 as an E3 Ligase

The steady state level of protein expression is a function of the rate of transcription/translation on the one hand and the rate of protein degradation on the other. The rate of protein synthesis is determined by different factors: firstly, ribosomal protein biogenesis, which is controlled by the rate of ribosomal RNA (rRNA) synthesis; secondly, mRNA synthesis and, thirdly, mRNA deadenylation. Protein degradation is dependent on the ubiquitin-dependent proteasomal degradation pathway. The Ccr4–Not complex influences all these steps, to a greater or lesser extent.

The production of ribosomes and ribosomal proteins is restricted by environmental nutrient levels. The Ccr4–Not complex, downstream of mTORC1, regulates the RNA Polymerase I (RNA Pol I)-dependent rRNA expression and couples nutrient signaling to the control of rRNA synthesis. Ccr4–Not localizes to rDNA and physically interacts with the RNA Pol I holoenzyme [123]. Co-translational association of the N-terminal domains of the proteasomal regulatory subunits Rpt1 and Rpt2 alleviates ribosome pausing. The N-terminal disordered domain of Rpt1 is important for efficient ribosome pausing and incorporation of nascent Rpt1 protein complexes into ‘heavy soluble particles’. Immunofluorescence and in situ hybridization studies have shown the co-localization of Rpt1 and Rpt2 mRNAs in these heavy particles that contain, and are dependent upon, Not1 [124]. These heavy particles or granules have been termed ‘Not1-containing assemblysomes’ as they stabilize ribosome-nascent chain complexes (RNCs) to enable co-translational association of nascent chains [124].

The RNA Pol II subunits Rpb4p and Rpb7p form a heterodimer, interact with Nip1 and Hcr1, two components of translation initiation factor 3 (eIF3), and stimulate translation initiation [125]. The interplay between Ccr4–Not and the Rpb4/7 module might play a role in imprinting of mRNAs during transcription for future deadenylation [126]. In budding yeast mRNA deadenylation is coupled to translation rates by the differential nuclease activities of the Ccr4–Not complex [41]. Efficiency of translation is determined during transcription elongation through the imprinting of mRNAs with Not1 [127]. Indeed, the cross talk between the yeast Ccr4–Not complex and Rpb4/7 links transcription and mRNA decay to translation.

**CNOT4/Not4-E3 ligase and its role in protein degradation**: The ubiquitin (Ub)-proteasome pathway (UPP) of protein degradation regulates cellular protein turnover. Protein destruction requires an ATP-dependent process that involves three enzymes: an E1 (ubiquitin activating), an E2 (ubiquitin conjugating) and an E3 (ubiquitin ligating) enzyme. E1 first binds to an ubiquitin molecule and transfers it to E2. E2-ubiquitin interacts with the E3 and ubiquitin is transferred to the substrate. The ubiquitylated proteins are recognized by the 26S proteasome intracellular protease [128,129].

CNOT4/Not4 is a RING finger E3 ligase and capable of auto-ubiquitylation [130,131]. The RING domain is located at the N-terminus of Not4 and is essential for Not4 ubiquitylation activity, but not for binding to the Ccr4–Not complex [130,132,133]. The CNOT4 RING domain preferentially interacts with the UBC4/5 subfamily of closely related human E2 enzymes both in vivo and in vitro [130], as well as the yeast orthologues Ubc4p and Ubc5p [131]. The ribosomal protein Rps7A, ribosome-associated chaperone NAC (nascent polypeptide associated complex in mammals), RNA Pol II subunit Rpb1, the demethylase Jhd2 and cyclin C are known yeast Not4 substrates [134,135,136,137,138,139]. The action of the Not4 E3 ligase on these substrates has obvious ‘downstream’ cellular consequences, such as an increase in H3K4me3 histone methylation due to the reduction in Jhd2, as we will discuss later in Section 5 [140].

A synthetic lethal screen first linked Not4 to the ubiquitin proteasomal pathway and, subsequently, genetic and biochemical studies confirmed that Not4 associates with the proteasome in vivo [131,140]. Cells lacking Not4 manifest the accumulation of polyubiquitylated, misfolded and aggregated proteins, decreased free ubiquitin levels and defects in proteasome integrity [133].

Association of Not4 with Ecm29, a proteasome chaperone, is important for the correct assembly of the proteasome, and co-translational quality control [17,133,141]. The ribosome-associated chaperone complex EGD (enhancer of Gal4 transcriptional activator) complex consists of the Egd1p and Egd2p subunits in *Saccharomyces cerevisiae* (NAC in mammals). NAC is an important component of the proteostasis network that assists with the folding of nascent proteins and functions as a sensor for downstream protein folding conditions. Both Egd1p and Egd2p are ubiquitylated proteins, whose ubiquitylation status is regulated by Not4 [135]. EGD ubiquitylation is important for its ribosome association and stability. In the absence of Not4p, Egd2p mis-localizes [135,136].

Ribosomal protein Rps7A, but not its paralogue Rps7B, is ubiquitylated by the Not4 E3 ligase. Ub-Rps7A is only present in the large 80S ribosomal subunit and polysomes and not in the free 40S ribosomal fraction [134]. Although Rps7A ubiquitylation is dependent on the Not4 subunit, and not any of the other components of the Ccr4–Not complex, Rps7A ubiquitylation is increased in cells containing mutant Not4 that cannot bind to the Ccr4–Not complex. This may suggest that the level of ubiquitylation activity of Not4 is regulated by the CNOT holocomplex [134].

## 5. The Ccr4–Not Complex and Transcription

**Figure 2:**
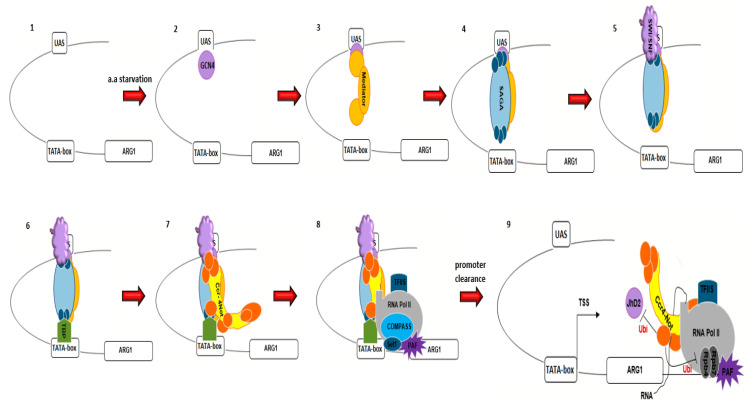
The presence of Ccr4–Not complex in pre-initiation and elongation complexes. In response to amino acid starvation, Gcn4 binds to the ARG1 gene promoter. The recruitment of Mediator, SAGA and SWI/SNF is nearly simultaneous; however, genetic evidence suggests that Mediator binding precedes SAGA and SWI/SNF, and that SAGA precedes SWI/SNF [228]. TBP is loaded and finally, Pol II recruited to the promoter. The timing of the Ccr4–Not complex loading to the core promoter is less clear. Promoter clearance preceded with the phosphorylation of the RNA Pol II CTD tail. The Ccr4–Not complex is also present in elongation complexes, which includes TFIIS and Pa1c. It contributes to elongation by inhibition of JhD2, and rescue of backtracked RNA Pol II.

The Ccr4–Not complex regulates transcript buffering in yeast [142]. It controls gene expression by altering both the transcription elongation rate and the rate of mRNA decay [143,144,145,146]. Importantly, the Ccr4–Not complex can both negatively and positively regulate global gene expression by affecting both transcription initiation and elongation activities. Importantly, the Ccr4–Not complex regulates both RNA Pol I and RNA Pol II transcription activities [3,123,137,147]) (Figure 2). 

The Ccr4–Not subunits associate with transcription regulatory factors on the 5’ regions of mRNAs, such as TFIID, the SAGA (Spt–Ada–Gcn5 acetyltransferase) histone acetyltransferase complex, TATA binding protein associated factor (TAF), TATA-binding protein (TBP), SRB/Mediator complex and PAF (RNA polymerase II-associated factor) complexes both genetically and physically, reflecting a role for the Ccr4–Not complex in transcription initiation as well as in elongation [51,148,149,150,151,152,153,154].

Initially it was shown that four yeast Not proteins were involved in the global repression of transcription initiation [11,155]. However, it now seems reasonable to suppose that the whole complex plays a role in the process [123,156]. The Ccr4–Not complex functionally and physically associates with TBP and TBP-associated components [152]. Specifically, TBP canonical binding is highly suppressed by Not1 in yeast [155,157]. Not1 has the ability to bind to TBP in the TFIID and SAGA regulatory transcription factor complexes, which function at approximately 90% and 10% of the gene promoters, respectively [158,159,160]. TFIID is typically found on promoters of constitutive TATA-less housekeeping genes that are required for the maintenance of basic cellular functions, while SAGA-dominated genes are largely stress induced and TATA-containing. 

**SAGA** is a chromatin-modifying complex that possesses two distinct enzymatic activities, acetylation of histones H3 and H2B via the Gcn5 histone acetyltransferase (HAT) and deubiquitylation of histone H2B via enzymatic activity of Ubp8 [161,162]. Indeed, the SAGA complex induces a pattern of histone modifications that is required for more effective TATA binding by TBP [162]. The SAGA complex consists of adaptor (Ada) and functional components (Spt and Gcn5) as well as several structurally important proteins such as HAT and BrD. The SAGA complex is proposed to interact with transcription activators (such as Gcn4 and GAL) at the upstream activation sequence through Ada2 [163]. The yeast Not5 protein contributes to co-translational assembly of the SAGA complex, which is important for its functional integrity [164]. In addition, the presence of Tdh3, a yeast glyceraldehyde 3-phosphate dehydrogenase (GAPDH), at the site of Spt20 production, is Not4- and Not5-dependent. Tdh3 functions as a chaperone and is also important for functional assembly of SAGA [164]. Ccr4–Not subunit crosslinking is enriched at SAGA/Gcn4-dependent genes, such as those involved in amino acid biosynthesis (for example, HIS3 (TR element), ARG1 and PYK1) and stress responses [148,165,166]. ARG1, PYK1, HIS3 encode enzymes that are involved in the arginine, pyruvate and histidine biosynthesis pathways, respectively. Genome-wide gene expression analysis of Ccr4–Not mutants has shown that SAGA-responsive genes are those that are largely affected [165]. Moreover, a chromatin immunoprecipitation-sequencing (ChIP-seq) study has confirmed the loading of Ccr4–Not components to the open reading frames of SAGA-regulated genes [167].

The role of Ccr4–Not and SAGA is illustrated by consideration of the yeast HIS3 promoter region that contains two functionally distinct TATA elements, constitutive (TC) and regulatory (TR). They can be distinguished by their interactions with upstream promoter elements, by their effects on the selection of initiation sites, and by their physical structure in nuclear chromatin [168]. TC is located between positions -83 and -53, an extended region of a cluster of weak non-consensus TATA elements that does not support transcription in vitro. In contrast, TR is localized between positions -55 and -35, and contains several canonical TATAAA sequences (TATA-like sequences). TC and TR are responsible for transcription initiation from the +1 and +13 nucleotides respectively [169]. TFIID is required for transcription from the non-conventional TATA sequence at the TRP3 promoter and the TC element of the HIS3 gene promoter. Whilst SAGA is responsible for transcription from TATA-containing regions such as the TR element of the HIS3 gene, TBP can bind to both TC and TR elements, with higher affinity to TC, and support basal transcription from HIS3. However, TR is the only element that can respond to transcriptional activators such as Gcn4 and GAL which are responsible for increased transcription activity [170]. The Ccr4–Not complex preferentially inhibits TC-dependent transcription, perhaps by interacting with, and modulating the function of, TBP [11,152]. A temperature-sensitive mutation in Not1 increases the basal transcription of HIS3 TC region [171]. The N-terminal region of Not2 associates with the Ada/Gcn5 of the SAGA complex. Loss of this interaction, either through disruption of the Not2 N-terminal region or deletion of Ada or Gcn5, results in increased basal transcription of HIS3 TC element [150,172]. 

SAGA-TBP interaction, is principally provided by SAGA coactivator subunit Spt8, in conjunction with Spt3 [173]. Spt3 mutations that affect its interaction with TBP suppress Ccr4, Caf1 and Not1 mutant defects [152,160,174]. Transcriptional activation observed in Not1 mutants correlates with increased binding of TBP in TFIID and SAGA at promoters of upregulated genes, and this is suppressed by the deletion of Spt3 [160]. Ccr4 gene deletion specifically suppresses the loss-of-function phenotype associated with the Spt5 mutant [175]. The heterodimer Spt4-Spt5 (DSIF) complex regulates mRNA processing and transcription elongation by RNA polymerase II [176]. 

The **SRB/mediator** that is associated with RNA polymerase II holoenzyme can act as both a positive and negative regulator of transcription [177,178]. A distinct sub-complex composed of the Med12, Med13, Cdk8, and cyclin C subunits has been implicated in transcriptional repression in *Saccharomyces cerevisiae* [179]. An interaction between Cdk8/cyclin C (Srb10/11), a kinase component of SRB/Mediator complex, and Ccr4–Not has been described [153]. In response to different physiological signals Cdk8/cyclin C binds to and/or phosphorylates at least four site-specific DNA-binding proteins (Gal4, Ste12, Gcn4, and Msn2), changing their activity by several mechanisms. The cyclin-kinase pair Cdk8 and cyclin C inhibits the expression of a subset of genes involved in the stress response [180]. Not4 ubiquitylates cyclin C and thereby marks it for proteasomal degradation in response to H_2_O_2_-induced oxidative stress [139]. Mutations in other Not genes suppress temperature-sensitivity associated with the mutated Srb4 [181].

In mammals, the Not-Box motifs of the CNOT2 and CNOT9 subunits can directly repress the association of RNA Pol II with TATA-box promoter sequences [182,183,184]. In yeast cells lacking Not4 or Not5, histones H3 and H4 are generally hypoacetylated [185]. Loss of NOT3 in knockout mice results in early embryo mortality due to heart failure. The heart defects can be reversed by inhibition of HDACs, indicating again the involvement of histone acetylation in the process [186]. Using a genome-wide RNAi screen human CNOT1 and CNOT2 were also identified among those proteins that contribute to repression of major histocompatibility complex class II transcription [187].

Different subunits of the Ccr4–Not complex exhibit variations in their effects on gene expression [188]. Additional support for such a suggestion was provided by the observation that yeast strains with mutations in Not1, Not2, Not4, and DBF2 increase the β-galactosidase reporter activity resulting from increased expression of hormone-dependent nuclear lacZ reporter genes such as HO–lacZ, FKS1–lacZ or FUS1–lacZ [189,190,191]. In contrast, strains containing deletion of Ccr4, Caf1 or Not3 show decreased β-galactosidase activity [189]. In other studies, it has been shown that overexpression of hCcr4 (CNOT6/6L) or hCaf1 (CNOT7/8) increases the ligand-dependent gene expression of the retinoic acid receptor (RAR) and oestrogen receptor α (ERα) in human cells [104,192]. In contrast, CNOT1 or CNOT2 suppresses the ligand-dependent transcriptional activation of ERα and retinoid X receptor (RXR) target genes, such as the proto-oncogene *c-Myc* in MCF-7 human breast cancer cells [182,183].

Overexpression of *c-Myc* stimulates the universal amplification of gene expression by all three RNA polymerases [193,194]. However, in human cells a decrease in the levels of *c-Myc* mRNA has been reported following depletion of CNOT9 [192]. CNOT3 and Trim28 (KAP1) co-occupy many putative gene promoter regions in close proximity to TSS with *c-Myc* and Zfx [109]. The observation that the Ccr4–Not complex can behave as both a repressor and as a co-activator for hormone-dependent transcription activity could be due to the use of different experimental systems or, perhaps more likely, reflect a gene- and promoter-specific function of each subunit in different species.

In other studies, it has also been shown that the same subunit can have varying effects in different experimental systems. For example; in asymptomatic carriers of retinitis pigmentosa, a group of rare, genetic disorders that involve a breakdown and loss of cells in the retina, low level expression of CNOT3 leads to higher level of the wild-type splicing factor PRPF31 mRNA, confirming a repressive regulation by CNOT3 on PRPF31 gene expression in human cells [195,196]. A deficient PRPF31 gene is one of several retinitis pigmentosa-causing genes (see Section 11). However, in mice CNOT3, but not other components of the Ccr4–Not complex, has been identified among those genes whose function contributes to the formation of a unique module in the transcriptional network required for self-renewal in embryonic stem cells [109].

The positive role of the Ccr4–Not complex in transcription activity is supported by evidence showing that the Ccr4 protein is present in a large cluster of proteins involved in transcription elongation by RNA Pol II at promoter regions [3,197]. This is consistent with the observation that yeast strains containing mutations in Ccr4–Not genes show synthetic lethality to transcription elongation inhibitors [175,198].

The interaction between Spt6, a histone chaperone, and RNA Pol II maintains chromatin structure during transcription elongation, thereby repressing transcription initiation by mediating the reassembly of nucleosomes onto the promoters [199,200,201]. The association between Spt6-RNAPII and Ccr4 has been confirmed [202]. Mutations in Ccr4 and Caf1 are known mutations that decrease the ability of Spt6 defects to allow enhanced expression of glucose-repressible alcohol dehydrogenase (ADH2) [62,203,204,205]. Mutations in the Not genes, except for Not3, decrease the expression of ADH2 under non-fermentative conditions, suggesting that the Not proteins are associated with positive control elements [189].

Not4 appears to contribute to different aspects of transcription from initiation to elongation. It indirectly controls histone H3K4 tri-methylation, an active marker of transcription, by two mechanisms: firstly, by regulating the activity of the PAF1 complex (PAF1C), on the one hand, by facilitating the loading of PAF1C on to promoters and, on the other hand, by ubiquitin-dependent proteolysis of PAF1 not bound to chromatin through the 26S proteasome [166,206]. Set1-directed methylation of H3K4 depends on the PAF1C-dependent ubiquitylation of H2B [207]. Secondly, by regulating the ubiquitin-dependent degradation of the demethylase Jhd2 [138]. Not4Δ cells show a significant reduction in the level of H3K4me3 [140,166]. It seems that this reduction in the level of H3K4me3 does not strictly correlate to a global decrease in recruitment of an RNA polymerase II holoenzyme to promoters but is limited to stress-responsive genes. For example, chromatin immunoprecipitation (ChIP) experiments reveal that the recruitment of TBP, RNA Pol II and Set1 to the activated RNR3 locus is diminished in Not4Δ cells [208]. Disruption of Not4 RRM-C (conserved RNA recognition motif and C3H1 domain) decreases the gene expression of transcription elongation factors Spt16 and Spt5; however, it increases the expression of genes involved in cell cycle control (PCL9, PCL5 and CLB1) and ribosomal biogenesis [209]. During elongation, removal of persistently stalled RNA Pol II at DNA lesion sites can be facilitated by Not4-mediated polyubiquitylation and proteasome-dependent degradation of the largest RNA Pol II subunit Rpb1 in *Saccharomyces cerevisiae* [137]. This requires the association between the Rpb4/7 module of RNA Pol II and both Not3 and Not5 [210]. It seems that the length of the nascent RNA is an important determinant in forward translocation of stalled RNA Pol II by the Ccr4–Not complex [7]. Transcription factor S-II (TFIIS) is another protein that directly induces resumption of elongation and helps RNA Pol II to pass template-encoded pause sites by increasing the intrinsic nuclease activity of RNA Pol II and displacing transcripts [211,212,213]). Both 5′–3′ exoribonucleases Xrn1 and Ccr4–Not regulate RNAPII elongation in parallel. By measuring TFIIS genome-wide recruitment to elongating RNAPII it was shown that Xrn1Δ and Ccr4Δ deletion mutants had different patterns of TFIIS and RNAPII occupancy, indicating distinct roles in regulating transcription elongation [145]. The ribonuclease activity of the Ccr4–Not complex does not appear to contribute to this mechanism; however, the Ccr4–Not complex improves the recruitment of TFIIS to elongation complexes and this is mediated by an interaction between the complex and the N terminal region of TFIIS [3,214,215]).

The similarity between the phenotypes of Ccr4–NotΔ and Rpb4Δ mutants suggests that they overlap in their roles in controlling gene expression and decay rates. For example, they both show increased sensitivity to the transcription elongation inhibitor 6-azauracil, impaired transcription through long GC-rich regions, impaired transcription-coupled repair, and decreased mRNA turnover [175,198,216,217]. In addition, in response to cellular stress both Rpb4/7 and Ccr4–Not subunits can localize to cytoplasmic foci [13]. The interplay between Ccr4–Not and Rpb4/7 may play a role in imprinting of mRNAs during transcription for future deadenylation [126]. As we will discuss in Section 8, Spt6 and RNA Pol II promote the recruitment of the Ccr4–Not complex at the site of transcription in order to facilitate the degradation of mRNAs required for cell-cycle progression [202]. Piwi-interacting RNA (piRNA) can also recruit nuclear Ccr4–Not at the site of transcription to degrade the expression of transposable element (TEs) transcripts on chromatin in the *Drosophila* germline [218]. Transcription from stress responsive genes is accompanied by opposing effects on their mRNA degradation, indicating the link between mRNA synthesis and degradation [143,219,220,221].

Transcription increases recombination rate by a phenomenon known as “transcription-associated recombination” (TAR) [222,223]. TAR is required for developmentally regulated processes, such as class switch recombination (CSR) and somatic hyper-mutation (SHM) of immunoglobulin (Ig) genes [224,225]. Transcription might play a role in initiation of CSR, by forming RNA:DNA structures [224,225,226,227].

## 6. A Potential Role for the Ccr4–Not Complex in Repair of Transcription-Dependent Replication Stress

**Figure 3:**
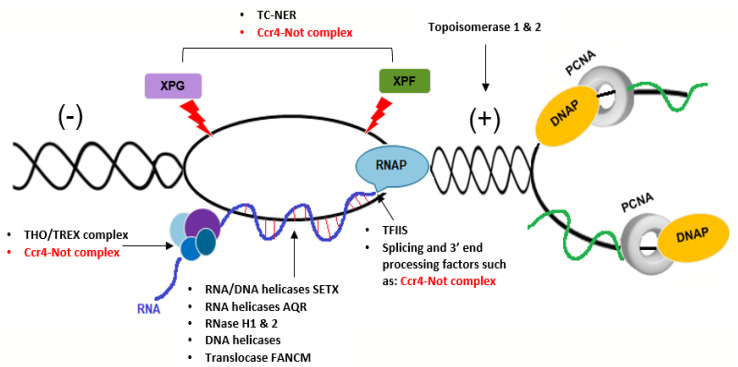
The potential roles for the Ccr4–Not complex in repair of R-loops. Three groups of surveillance factors are involved in repair of R-loops; the first group, such as THO/TREX complex, binds to nascent RNA and prevents the hybridization between the ssDNA template and the nascent RNA. The second group, such as topoisomerases, co-transcriptionally contributes to the overall stability and processivity of RNA Pol II splicing and 3′ end processing factors. The final group actively removes the R-loop structures; for example, the RNA/DNA helicases such as Senataxin (SETX) and/or Aquarius (AQR), DNA helicasetranslocase FANCM and RNase H enzymes. We suggest that the Ccr4–Not complex can contribute to function of all these factors as shown.

Transcription may affect DNA replication if co-transcriptional products such as R-loops (three-stranded DNA–RNA hybrid structures) are not efficiently removed behind the transcription machinery. The collision of replication and transcription complexes with R-loops threatens genome integrity by increasing the rate of mutagenesis, particularly over highly active promoters, such as those that occur in ribosomal genes (rDNA) [229,230]. Genome stability is protected against R-loop formation via three sets of surveillance factors: the first group co-transcriptionally contributes to the overall stability and processivity of RNA Pol II, and includes TFIIS, topoisomerases, splicing and 3′ end processing factors [231,232,233,234]. The second group binds to nascent RNA and prevents RNA hybridization with the ssDNA template and this includes the THO/TREX complex. The third group actively removes the R-loop structures; for example, the RNA/DNA helicases such as Senataxin (SETX) and/or Aquarius (AQR), the DNA helicase/translocase FANCM, and RNase H enzymes [235,236] (Figure 3).

TFIIS directly induces the resumption of elongation and helps RNA Pol II to pass through trapping sites by increasing the intrinsic nuclease activity of RNA Pol II and displacing transcripts [237]. As discussed in Section 5 the Ccr4–Not complex functionally co-operates with TFIIS to rescue arrested RNA Pol II. Expression of mutant TFIIS has been shown to increase the levels of RNA Pol II pausing, and R-Loop formation in human cells [233]. Chromatin modelers, such as the SWI/SNF complex, which increases the accessibility of DNA to transcription factors, contribute to the TFIIS-dependent rescue process [238,239].

The THO/TREX complex is able to suppress R-loop formation by binding to the transcript or assembling hnRNPs onto the RNA, thereby preventing RNA-DNA hybrid formation [240,241,242]. Deletion of the yeast THO complex components, Tho2, Hpr1, Mft1, and Thp2, results in hyper-recombination phenotypes, transcription elongation impairment and increased formation of R-Loops behind the transcription machinery [243,244,245]. Elimination of the hyper-recombination phenotype in Hpr1Δ cells by overexpression of RNase H1, which resolves R-loop structures, indicates that these are formed co-transcriptionally [246,247]. Similarly, yeast mutants in the hnRNP subunit Npl3 show R-loop-dependent hyper-recombination, which impairs DNA replication. Rad52 foci formation in response to DSBs could be partially, but significantly, suppressed by RNase H1 overexpression in Npl3Δ cells. As we will discuss in more detail in the next section the Ccr4–Not subunits have been shown to physically interact with hnRNPs and the THO/TREX complex component Thoc5 [7,147,218,248]. The interactions between the Ccr4–Not complex and R-Loop processing factors indicate a role for the CNOT complex in repair of R-loop structures.

Transcription-coupled repair (TC-NER) nucleases XPG and XPF can excise R-loops that block transcription, leaving a ssDNA gap which can collapse into DNA DSBs [249]. TC-NER is impaired by mutations in the Ccr4–Not complex in yeast, suggesting additional functions in DNA repair during transcription elongation [198]. For example, R-loops are the main source of DNA damage following induction of the oestrogen (E2)-dependent transcription response [250]. CNOT1 directly interacts with the ligand-binding domain of ERα and represses the transcription of E2-regulated promoters in a ligand-dependent manner [183].

Mutations and agents that affect transcription elongation or mRNA processing can be detected by the GLAM reporter system [251]. Ccr4–Not complex mutants have been identified which impair transcription elongation through long GC-rich genes (GLAM phenotypes) with different GLAM ratios [198]. The long-GC-rich regions impede transcription elongation by forming R-loops, suggesting that Ccr4–Not promotes elongation through GC-rich regions by preventing R-loop formation during transcription [147].

While R-loops are a known source of genome instability they can also contribute to developmentally regulated processes, such as immunoglobulin (Ig) class switch recombination (CSR). CSR is needed for antibody maturation by an activated B cell. Interestingly, CNOT3 contributes to early B cell differentiation. In CNOT3-null mice, there is a block in the transition from pro-B (the phase where IgH class switch recombination occurs) to the pre-B stage (that express the pre-B cell receptor (pre BCR)) during the early B-cell development [50]. In addition, CNOT3 interacts with transcription factor EBF1 and enhances EBF1 occupancy at a specific set of target genes, including VpreB1 and Igll1 (λ5), components of the pre-BCR [252].

## 7. The Role of the Ccr4–Not Complex in the Export of Nascent RNA through Nuclear Pores

The Ccr4–Not complex is strongly associated with the mRNA export system, which bridges the production of mRNA in the nucleus to its translation and degradation in the cytoplasm; in addition, the complex is important in nuclear RNA surveillance. This raises the possibility that Ccr4–Not may remain bound to the mRNA during nuclear export. The CNOT complex and BTG heterodimer have been reported to associate with, and regulate, the arginine methyltransferase PRMT1 in a substrate-dependent manner [253,254,255,256]. BTG1 and BTG2 can also interact with the mammalian Ccr4–Not complex [56,104,257]. PRMT1 targets RNA binding proteins (RBPs) that are involved in mRNA export, such as hnRNPs and Sam68 [258,259]. Ccr4–Not subunits physically and functionally interact with hnRNP methyltransferase Hmt1, the budding yeast orthologue of PRMT1 [248]. The methylation of the yeast hnRNPs Npl3p and Hrp1p and Sam68 is pivotal for their cellular localization [259,260,261]. In addition, the physical association of Ccr4–Not subunits with hnRNPs Nab2 and Hrp1 depends on methyltransferase activity of Hmt1 [248]. These interactions are consistent with a significant role for the Ccr4–Not complex in mRNA export.

Several nuclear exosome subunits and their co-factor TRAMP complex have been found to co-purify with the Ccr4–Not complex [262]. The TRAMP complex polyadenylates the 3′ ends of aberrant noncoding RNAs. This poly (A) tail serves as the docking site for loading of the nuclear exosome to initiate RNA turnover [263,264,265]. The nuclear exosome is capable of degradation of various types of aberrant RNAs such as mRNA, rRNA and many species of small RNAs using its 3′ to 5′ exonuclease activity [266,267,268]. The genetic depletion of Ccr4–Not subunits gives rise to synthetic growth phenotypes when combined with the deletion of exosomal proteins, suggesting collaboration between the two complexes [262,269]. For example, it has been demonstrated that mutations in Ccr4–Not subunits lead to the accumulation of polyadenylated and misprocessed snRNA, giving rise to phenotypes similar to those seen with exosomal Rrp6 mutants [262]. In the fission yeast *Schizosaccharomyces pombe*, a nuclear isoform of the CNOT complex interacts with the RNA-binding protein Mmi1 and promotes the exosome-mediated elimination of meiotic transcripts during vegetative proliferation [8,24,270,271]. 

As mentioned previously, the Ccr4–Not complex also interacts with Thoc5, a component of the human THO/TREX mRNP packaging complex [7,147,218]. Similarly, mutation of the Ccr4–Not complex components and the deletion of the yeast THO complex components Hrp1 and Mft1 is synthetically lethal, which suggests a functional relationship between the two complexes [245,269]. The overexpression of Not4 in nuclear pore complex (NPC) mutants exacerbates the poly (A) mRNA export defect, suggesting altered ubiquitylation of NPC or RNA export factors may be responsible for this phenotype [248]. Indeed, Not4 may increase the ubiquitylation-dependent degradation of NPC or RNA export factors or interfere with normal NPC function by binding to RNAs through its conserved RNA recognition motif [6,130].

## 8. The Role of the Ccr4–Not Complex in Cell Cycle Regulation

The CNOT/Ccr4–Not complex affects cell cycle progression through several different mechanisms in both mammalian and yeast cells. Many proteins regulate the timing and frequency of DNA duplication and cell division during the cell cycle. A novel role for the Ccr4–Not protein complex in the regulation of mRNAs involved in cell cycle progression in yeast has been demonstrated [202]. These authors have shown that the interaction between Spt6, a histone chaperone, and RNA Pol II promotes the recruitment of the Ccr4–Not complex at the site of transcription in order to facilitate the degradation of mRNAs required for cell-cycle progression. The stability of HTA1 (mammalian H2A) and CLB5 (cyclin B) mRNA poly (A) tails increases in Spt6 (spt6tSH2Δ) and Ccr4Δ mutant strains compared with wild-type [202]. This recruitment and activity of the Ccr4–Not complex probably occurs during the G1 phase when the complex is known to be concentrated in the nucleus [56]. It has previously been established that much of the complex becomes cytoplasmic during S phase and is, therefore, no longer available. This is consistent with flow cytometric analysis which has revealed that depletion of CNOT1 delays cell cycle progression by inhibiting S phase transition in osteosarcoma cells [272]. Similarly, depletion of CNOT1, CNOT3, CNOT6 and CNOT6L in MCF7 cells reduces cell proliferation, seen as a decreased fraction of cells in S phase, and an increase of cells in G1 [273]. Similar to E26-related gene (ERG) siRNA-treated cells, depletion of CNOT2 inhibits mitotic progression of HeLa cells by stabilization of most of the mitotic ERG-target mRNAs, such as cyclin B1 [144]. ERG also promotes mRNA decay of Aurora signaling components, whose degradation during S phase is required for mitotic progression [144]. 

Twin, the homologue of the yeast Ccr4 deadenylase, regulates the length of cyclin A poly(A) tails to permit *Drosophila* *melanogaster* oogenesis [114]. In the *Drosophila* embryo, maternal cyclin B mRNA in the germline is directly repressed by binding of RNA-binding proteins Nanos2 and Pumilio to an element in its 3′ UTR. Nanos2 is responsible, at least in part, for recruiting the Ccr4-Pop2-Not deadenylase complex, interacting directly with the Not4 subunit [274]. However, in *Xenopus* oocytes, the shortening of cyclin B mRNA is directed and controlled by cytoplasmic polyadenylation element binding protein (CPEB), poly (A)-specific ribonuclease (PARN), and embryonic poly (A)-binding protein I (ePAB I) [275,276]. Mammalian oocyte maturation depends on the translational activation of a subset of maternal mRNAs upon meiotic resumption. CPEB1 is a key oocyte factor that regulates maternal mRNA translation [277]. During oocyte maturation, CPEB1 triggers the translation of some key CPE-containing maternal mRNAs, such as those of CNOT7, BTG4, and cyclin B1, in a MAPKERK1/2-dependent manner [278,279,280,281]. CNOT6L-dependent decay of selective maternal mRNAs is a prerequisite for mouse oocyte meiotic maturation. CPEB1 mRNA was among transcripts stabilized in CNOT6L−/− mice leading to over-translation [282]. As a result, the microtubule–chromosome organization impairs, and leads to activation of, the spindle assembly checkpoint and metaphase II cell cycle arrest [282].

The transcriptional profile analysis of yeast cells containing mutant Not4 RRM-C (conserved RNA recognition motif and C3H1 domain) has shown a reduction in cyclins PCL9, PCL5 and M-phase cyclin CLB1 (functional homologues of mammalian cyclin B) [209]. Cyclin PCL9 in complex with the cyclin-dependent kinase Pho85 is expressed in late M/early G1 phase and may play a role in bud site selection in G1 phase [283,284]). However, PCL5-Pho85 seems to function in amino-acid biosynthesis by regulation of the transcription factor GCN4 [285,286]. Pho85 and other G1 Cdks (CLN1-3) appear to phosphorylate the Cdk-inhibitor SIC1 which inhibits the Cdc28-CLB kinases at different sites. SIC1 phosphorylation triggers its degradation in G1 cells and initiates DNA replication [287]. SIC1 binds to the Cdc28-CLB (CLB1-6) kinase complexes and regulates the timing of M/G1 and G1/S cell cycle transition, by inhibiting their activities [288,289,290,291].

In an early study it was demonstrated that, during telophase/G1 phase transition, Dbf2, which is a cell cycle-regulated Ser/Thr protein kinase and a possible member of a larger Ccr4–Not complex, is involved in transcription of genes required for cell cycle progression [292]. Dbf2p also controls the cell cycle-regulated decay of SWI5 and CLB2 (mammalian cyclin B) mRNAs [293]. SWI5 transcription factor positively increases the expression of SIC1 that promotes mitotic exit [288]. It had previously been shown that the SICl gene interacts genetically with Dbf2, and that SICl gene expression is under cell cycle control in telophase to early G1 [294]. SIC1 is a functional homologue of mammalian p27Kip1 [295]. It has been reported that the level of mammalian p27, increases in mouse NIH 3T3 cells following depletion of CNOT6L [65]. This is due to an increase in the stability of p27 mRNA poly (A) tail. The highest expression levels of p27 occur during the quiescent G0 and pre-replicative G1 phases. Quiescence is mediated preferentially by p27-dependent Cdk inactivation and cyclin D1 downregulation. In quiescent cells, p27 inhibitors miR-221 and miR-222 are hypoactive towards p27-3′ UTR, leading to accumulation of p27 and cell cycle arrest. Ubiquitously expressed RNA binding protein Pumilio-1 (PUM1) interacts with p27-3′ UTR and induces a local switch in RNA structure that favours binding of miR-221 and miR-222 with efficient inhibition of p27 expression, leading to rapid entry to the cell cycle [296]. A direct interaction between PUM1 and CNOT7 had previously been reported [297]. Dominant-negative mutants of CNOT7 and CNOT8 decrease Pumilio-mediated repression. 

Cyclin G2 is an unconventional cyclin, highly expressed in post-mitotic cells. Unlike classical cyclins that promote cell cycle progression, increased cyclin G2 expression inhibits cell cycle entry and contributes to maintaining the quiescent state of terminally differentiated cells [298]. The knockdown of CNOT7 and CNOT8 increases the mRNA stability of PMP22 (growth arrest-specific protein 3) and, cyclin G2 [59].

Yeast G1-specific cyclins CLN1, CLN2, and CLN3 (functional homologues of mammalian cyclin E) in complex with Cdc28 (Cdk1) are required for G1/S transition at the START point [291]. In post-START phase (budded phase), Cdc28-CLB5 and -CLB6 (functional homologues of mammalian cyclin A) promote DNA replication in S phase. It has been suggested there is a partial functional redundancy between CLB5 and CLN genes [290]. CLN1 and CLN2 mRNA expression is delayed in yeast cells lacking Ccr4, which modulates the half-life of Whi5 mRNA, a transcriptional repressor of CLN1 and CLN2 [299]. Activation of protein kinase C1 (PKC1) is essential for cell cycle progression [300]. PKC1 is activated by Cdc28-CLN1/CLN2 at the G1-S transition at the START point [301,302]. PKC1 regulates the MAP kinase 1 (Mpk1), whose activity is stimulated at the G1-S transition of the cell cycle. The RNA pol II complex containing Cdc73, Paf1, Ccr4, and Hpr1 functions, at least in part, to transmit signals from the PKC1-Mpk1 kinase cascade to target a subset of yeast genes involved in both bud emergence and the mating pathway [154]. Activation of the CLN-Cdc28 kinase by overexpression of CLN2 significantly decreases the mRNA induction of the mating-specific gene FUS1 [303]. The mating pheromone response pathway is a signal transduction pathway that performs an essential role in mating between the two yeast mating types a and α [304]. Mating pheromones arrest the cell cycle in late G1 at the START point via the pheromone-activated FUS1 gene [305,306]. Not1 (CDC39) or Not2 (CDC36)-deficient yeast strains activate expression of the pheromone-inducible gene FUS1 [190,191], suggesting, at least partial, regulation by the Ccr4–Not complex.

Down-regulation of canonical histone proteins (H3, H4, H2A, H2B), which are responsible for packaging of newly synthesized DNA, disrupts cell cycle progression through S phase and beyond [307]. It has been reported that depletion of CNOT1, CNOT2, or CNOT3 in mouse ESCs down-regulates the expression of genes, which are involved in histone assembly and chromatin modification [108]. Alabert and colleagues, using the nascent chromatin capture (NCC) technique, have shown that mammalian CNOT1 was among those proteins enriched in nascent, newly synthesized chromatin [308]. Chromatin re-modelers are also required for regulation of cell cycle progression and gene expression. For example, at the G1/S transition, histone acetylation is required for the specification and activation of replication origins [309,310]. Histone acetylation of H3 is essential for cell cycle progression, and histone H3 serves as one of the major targets for Gcn5-containing histone acetylation complexes in vitro [311]. Cells lacking Gcn5 show a cell cycle defect and accumulate in G2/M phase [312]. Global H3 acetylation levels have been shown to be reduced in Ccr4Δ, Not4Δ and Not5Δ cells in *Saccharomyces cerevisiae* [185]. One reason for this reduction is that the physical interaction of the Ccr4–Not complex with the SAGA histone acetyltransferase complex regulates its activity directly [313]. 

Another relevant point is that the 3′ UTR RNA binding proteins Puf5/Mpt5, involved in Ccr4–Not-mediated deadenylation in yeast, are recruited to the mRNAs of many chromatin regulators, including those of the two major histone deacetylases Hda1 and Rpd3 [314,315,316]. Therefore, disrupting the Ccr4–Not complex could stabilize mRNAs of Hda1 and Rpd3, leading to an increase in their protein levels. 

Methylation of histone H4 arginine 3 by PRMT1 is essential for the establishment and retention of both histones H3 and H4 acetylation patterns across the β-globin locus during erythroid differentiation [317]. Human Caf1 (CNOT7) associates with, and regulates, PRMT1 activity in a substrate-dependent manner [254]. Depletion of hCaf1 in MCF-7 cells significantly modulates the methylation of endogenous PRMT1 substrates such as histone H4 and RNA binding protein Sam68 [254]. 

## 9. The Role of the Ccr4–Not Complex in Senescence, Apoptosis and Autophagy

Following irreversible DNA damage or other cellular triggers, cells either adapt to stress through a permanent cell cycle arrest (senescence) or are eliminated through programmed cell death (apoptosis) or the lysosomal degradation pathway (autophagy) [318,319]. The Ccr4–Not complex can influence all these processes.

### 9.1. Senescence

One of the most significant findings in modern cell biology was the observation that primary mammalian cells have a limited, very reproducible lifespan in culture under defined conditions [320,321]. After a certain number of population doublings cells become quiescent and go into what was termed ‘replicative senescence’ in the G1 phase of the cell cycle. In senescence, as it is generally known, cells are still viable and metabolically active but fail to go through the cell cycle or divide [322]. The Ccr4–Not complex appears to have a role in the regulation of senescence and apoptosis (see below) in mammalian cells. For example, up-regulation of cellular senescence via a p53-dependent pathway has been reported in MCF7 cells upon depletion of CNOT6/CNOT6L [273].

Cellular senescence is marked by increased DNA content and reduced histone synthesis, particularly in terms of the nucleosome histones H3 and H4 and the linker histone H1, as their expression predominantly occurs in S phase, [323,324,325]. Histone modifications, such as methylation, acetylation, and phosphorylation, show different landscapes in senescent cells and this is, at least partially, attributable to the Ccr4–Not complex. Different variant histone proteins control the dynamic changes occurring in chromatin [326]. A genome-wide study of senescent chromatin showed that more than 30% of chromatin is dramatically reorganized in senescent cells, including the formation of large scale domains of H3K4me3- and H3K27me3-enriched “mesas” over lamin-associated domains (LADs), as well as large H3K27me3-depleted “canyons” outside of LADs [327]. It is notable that the CNOT4/Not4 indirectly regulates histone H3 methylation through proteasomal degradation of Jhd2 demethylase [138,140,166].

In addition, portions of chromatin fragments are exported from the nucleus for autophagy/lysosomal degradation during senescence, which results in greater chromatin disorganization and disruption of chromatin–lamin interactions [328]. Lamins provide structure to the nucleus and are also involved in transcriptional regulation [329]. Involvement of lamins in accelerated cellular senescence has been reported by several studies [327,330,331]. Significantly, CNOT1 interacts with LMNA (lamin A) in osteosarcoma cells and depletion of CNOT1 suppresses the cell proliferation through the Hedgehog signaling pathway via its association with LMNA [272].

### 9.2. Programmed Cell Death/Apoptosis

It is now clear that there are a number of mechanisms by which cells can die [332]. The primary pathways are through intrinsic or extrinsic apoptosis, although others such as parthanatos and necroptosis have been described [332]. Relevant to this review, it has been shown that a sub G1 (i.e., apoptotic) fraction is markedly increased in CNOT1-depleted HeLa cells. Indeed, CNOT1 depletion increases CHOP (also known as GADD153 and DDIT3) mRNA levels and leads to activation of caspase-4, which is associated with endoplasmic reticulum (ER) stress-induced apoptosis [35]. Caspase-4 is generally considered to be a member of the inflammatory caspase group and is involved in pyroptosis (inflammatory caspase-dependent programmed cell death) [333]. Similarly, another study has shown that CNOT2 depletion also increases the levels of CHOP mRNA suggesting that induction of ER stress causes intrinsic apoptosis in a caspase-dependent manner [83]. Data from TUNEL assays has indicated that depletion of mouse Caf1 (hCNOT7) results in germ cell apoptosis mainly at the spermatid stage in young adult male mice [60,61]. In addition, data from flow cytometry studies confirms induction of apoptosis following depletion of Ccr4a (hCNOT6) and Ccr4b (hCNOT6L) in MCF7 cells [273]. CNOT3 deficiency induces p53-mediated apoptosis of pro-B cells by increasing the mRNA stability of p53. In this case CNOT1 and CNOT3 appear to associate with 3′-UTR of p53 mRNA [252]. At the post-transcriptional level, Pumilio 1 (PUM1) binds to mRNAs representing 1527 genes, with significant enrichment for mRNAs involved in signaling pathways, such as cell cycle and MAP kinase pathways, leading to their degradation. In particular, eight mRNAs promoting activation of p53 are suppressed by PUM1. Deleting PUM1 causes the strong activation of p53, leading to cell-cycle arrest and apoptosis mainly in spermatocytes; this contributes to lower sperm production and infertility. Post-transcriptional activity of PUM1 is mediated by the deadenylase module of the CNOT complex [297].

Strains of *Saccharomyces cerevisiae* lacking Ccr4 exhibit typical markers of apoptotic phenotypes such as enhanced cellular reactive oxygen species (ROS) levels, externalization of phosphatidyl serine, chromatin fragmentation, and increased caspase gene expression, suggesting that longer poly (A) tail length of the accumulated mRNAs in general contributes to enhanced cell death [334]. 

### 9.3. Autophagy

The phenomenon of autophagy has been recognized for well over half a century. Its most obvious phenotype in mammalian cells is the presence of large vacuoles present in the cytoplasm of autophagic cells [335,336]. The mechanism by which this occurs was unclear until a detailed analysis of autophagy in yeast was published throughout the 1990s [337]. It was shown that autophagy was dependent on a complex pathway comprising a set of Apg proteins (later termed ATG proteins), of which approximately 35 have been confirmed in yeast [338]. 

Evidence in support of the role of the Ccr4–Not complex in the regulation of autophagy in yeast first came from the observation that Caf1 was associated with Dhh1, a component of the P-bodies [339]. A more recent study has shown that Not1/CNOT1 interacts with the Dhh1 or its the mammalian homolog DDX6 and stimulates its ATPase activity. Dhh1 is a post-transcriptional repressor of autophagy, which acts by, at least partly, binding and inhibiting the expression of a set of ATG genes, including ATG3, ATG7, ATG8, ATG19, ATG20, ATG22, and SNX4/ATG24 [121]. Loss of CNOT1 or CNOT3 results in autophagy and cell death in mouse cardiomyocytes [340]. CNOT3 binds to the poly (A) tail of ATG7 mRNA and regulates its stability and ultimately level of protein expression. In hearts with depleted CNOT3 expression of the ATG7 was elevated [340]. 

The Ccr4–Not complex may function as an effector of the Ras/cAMP pathway that negatively controls the transcription from stress-regulated promoters, by preventing activation of transcription factor Msn2 [159,341]. Msn2 is required for transcription of STRE-containing promoters, such as the autophagic protein ATG8 [342]. Depletion of CNOT2 in H1299 cells leads to induction of the autophagy adaptor protein p62/SQSTM1 and LC3B-II conversion, with impaired autophagic flux [343].

The extracellular signal-regulated kinases (ERK1/2) signaling pathway regulates expression of autophagy and lysosomal genes [344,345,346]. The ERK1/2 Kinases are important effectors of the MAPK signaling pathway [347]. The RAS-RAF-MEK-ERK signaling cascade is hyperactivated in a variety of tumors [348]. ERK1/2 are activated by RAF kinase and inhibited by Dusp6 phosphatases. The RNA binding proteins PUM2 and TTP are reported to function in the post-transcriptional repression of Dusp6 mRNA, presumably in a CCR4–NOT-dependent mechanism [349,350,351].

The link between oestrogen receptor (ER) expression and autophagy has been reported in a number of different cancers [352,353,354]. ERα contributes to tumorigenesis by stimulating aberrant cell proliferation. ERα-mediated autophagy is associated with the generation of ROS, activation of ERK1/2, and reduction in the dNTP pool, leading to decreased survival rate of cancer cells [353,354,355]. CNOT1 can interact with the ligand-binding domain of ERα in a hormone-dependent manner and, together with the other Ccr4–Not subunits, is recruited to endogenous oestrogen-regulated target promoters such *c-Myc*, where it inhibits their expression as discussed in Section 5 [183].

Autophagy functions as a survival response for hypoxic cells through removal of damaged cellular compartments and recycling of components [356,357]. As we will discuss in Section 10 the Ccr4–Not complex plays a role in the response to low oxygen conditions. Also, the suppression of the ribonuclease reductase (RNR) complex, which provides dNTP precursors for DNA synthesis, leads to the induction of autophagy [355]. The yeast Ccr4–Not complex is required for activation of the RNR subunits in response to HU-mediated replication stress (as discussed below). 

## 10. The Ccr4–Not Complex and the DNA Damage Response

Each cellular genome is subjected to many thousands of DNA lesions per day, arising from both endogenous and exogenous environmental sources. In order to maintain the integrity of the genome cells have evolved mechanisms known collectively as the DNA damage response (DDR). The DDR employs multiple protein pathways to monitor and repair various types of DNA damage [358]. Screening of a genome wide *S. pombe* haploid deletion library for mutants sensitive to DNA damaging agents identified novel genes involved in DNA repair, in checkpoint response pathways as well as in the establishment and maintenance of chromatin architecture (including Gcn5, Spt3, and Spt5 components of the SAGA complex), transcription (including Caf1, Ccr4, and Rcd1 subunits of the Ccr4–Not complex), and microtubule related processes (including the DASH complex) [359].

Yeast repair genes, including Rad9, Rad52, Rad6, Rad27, and MUS81, have been shown to interconnect genetically and/or physically, in an ionizing radiation (IR)-mediated damage response network, with Ccr4 [360]. IR-sensitive diploid deletion of different subunits of the Ccr4–Not complex appears to affect the G1/S phase transition by delaying Cdc28-dependent start function, resulting in the production of large cells. This study suggested that Ccr4 is a member of the Rad9 checkpoint pathway and its deletion leads to an increased irradiation-induced lethality as well as G1 cell cycle arrest, as do defects in the Rad9 gene. Cdc28/CLB complexes are needed for Rad9 phosphorylation [361]; phosphorylation of Rad9 on T125 and T143 residues by Cdc28 promotes the interaction between Rad9 and Chk1 and the recruitment of the Rad9/Chk1 complex at the site of damaged DNA [361,362]. As discussed earlier, the Ccr4–Not complex indirectly regulates the expression of SIC1, an inhibitor of Cdc28 (Section 8).

In further studies Ccr4 and Chk1 have also been shown to act in the same epistatic pathway with respect to the response to HU-induced DNA replication stress [363]. In mammalian cells depletion of hCCR4 (CNOT6) results in a marked resistance to cisplatin-mediated apoptosis following induction of Chk2 phosphorylation on T68, while overexpression of CNOT6 impairs phosphorylation of Chk2 on T68 and increases the sensitivity of cells to cisplatin and to bleomycin [364]. Notably, the overexpression of hCCR4 has no effect on the activation of Chk1 (S317 or S345).

CRT1, a DNA binding protein, is phosphorylated by the yeast Mec1-Rad53-Dun1 DNA damage sensor complex following HU-induced replication stress. After activation of CRT1, it recruits the general transcription repressors Ssn6 and Tup1 to the promoters of the HU-inducible genes: ribonuclease reductase 2 (RNR2), RNR3 and RNR4, subunits of the yeast RNR complex [365,366]. CRT1 mRNA stability and its protein abundance are regulated by Ccr4 [363]. The yeast RNR complex consists of two large catalytic subunits RNR1 or RNR3 (SpCdc22) located in the cytoplasm and two distinct small regulatory subunits RNR2 and RNR4 (SpSuc22) in the nucleoplasm. In response to DNA-replication stress Caf1 interacts with Suc22 and promotes the degradation of the RNR inhibitory protein Spd1 that holds Suc22 in the nucleoplasm resulting in release of Suc22 to form the active RNR complex in the cytoplasm [367]. In addition, the yeast Ccr4–Not complex is required for transcription activation of the RNR subunits by facilitating the recruitment of TBP, RNA pol II and the methyltransferase Set1p to the promoter of RNR3, following replication stress and DNA damage [208]. RNR catalyses the conversion of ribonucleotides into deoxyribonucleotides by removing the 2’-hydroxyl group of the ribose ring of the nucleoside diphosphates. The mammalian RNR is a tetrameric protein complex and consists of two catalytic (RRM1) and two regulatory (RRM2, RRM2B) subunits [368]. Reduction in RRM2 disrupts the activity of the RNR complex leading to depletion of the dNTP pool and retardation of DNA replication [369]. In response to HU-mediated replication stress the Not4 E3 ubiquitin ligase modulates the Ubc4p/Ubc5p-mediated stress responses [131]. RING-finger mutant Not4L35A-mediated HU sensitivity is independent of defects in RNR gene transcription. Moreover, phospho-Not4 functions in parallel with the BUR2 kinase in tolerance to HU-induced replication stress. However, it has been shown that BUR2 is not required for phosphorylation of Not4 [370]. 

Mammalian target of rapamycin complex 1 (mTORC1) is a master regulator of cell proliferation, autophagy and apoptosis by participating in multiple signaling pathways [371]. Reduced mTORC1 activity has been found to increase the level of the pro-apoptosis- and hypoxia-related protein NDRG1 (N-myc downstream-regulated gene 1). NDRG1 binds and stabilizes MGMTs (methyltransferases, chiefly O^6^ -methylguanine-DNA methyltransferase), key enzymes in the alkyl transfer repair mechanism and in resistance to cisplatin-mediated apoptosis [372]. CNOT1 and CNOT3, downstream of mTOR1 signaling, negatively regulate the transcription of DNA repair enzymes NDRG1 and MGMT. Consistent with this, reduction in CNOT1 and CNOT3 expression in three varieties of long-lived mice enhances expression of NDRG1 and MGMT proteins [373,374]. 

Use of a CRISPR-Cas9-mediated knockout HAP1 cell line, with individual mutations in five Fanconi Anemia (FA) genes, has shown that CNOT1 was among those genes whose inactivation reduces the sensitivity to Mitomycin C (MMC)-mediated DNA damage [375]. Similarly, the use of a genome-wide screen in yeast has allowed the identification of genes, including Ccr4, whose deletion confers sensitivity to cisplatin and MMC [376].

The homeodomain-interacting protein kinases (HIPKs) enhance apoptotic functions in response to various stress signals, including DNA damage, reactive oxygen species, and hypoxia [377]. CNOT2 and CNOT3 have been identified as interaction partners for HIPKs through their NOT-boxes [49]. Of interest, the yeast YAK1 kinase, a phylogenetic ancestor of the HIPK family, associates with Not1 and phosphorylates POP2/CAF1 (hCNOT7), upon glucose starvation [378]. Significantly, camptothecin-triggered cell death is regulated by HIPK2 and CNOT2 [49].

The Ccr4–Not complex is intimately involved in the cellular response to hypoxia. Transcriptional profiling analysis of a ΔCcr4 mutant in *C. albicans* revealed an interconnection with the early response to oxygen deprivation (hypoxia), suggesting a role for Ccr4 in low oxygen conditions. This response may be initiated by a drop in ATP generation [379]. The mitochondria of the ΔCcr4 mutant are compromised with an abnormal morphology, leading to hypoxic growth, even under normoxic conditions [380]. Hypoxic cells are characterised by reduced DNA repair, an increased mutation rate and increased chromosomal instability. 

Paradoxically, acute hypoxia induces production of reactive oxygen species (ROS), in the form of H_2_O_2_, for example [381]. Not4 ubiquitylates cyclin C and thereby marks it for proteasomal degradation in response to H_2_O_2_-induced oxidative stress [139]. The cyclin-kinase pair Cdk8 and cyclin C inhibits the expression of a subset of genes involved in the stress response [180]. Srb10 plays a key role in the regulation of the oxidative stress transcription factor Skn7 in a Not4-dependent manner [382]. Hypoxia induces H3K4me3 by inhibition of JARID1A (KDM5A) demethylase [383]. CNOT4 indirectly regulates the levels of H3 K4 trimethylation by polyubiquitylation of JARID1C (KDM5C) [138]. *Saccharomyces cerevisiae* Yap1 is a transcriptional regulatory protein that activates the expression of genes required for oxidative stress tolerance [384,385]. Not4 has been shown to bind to, and be involved in the degradation of, Yap1 in an oxidant-induced manner. Not4Δ yeast cells exhibit Yap1-mediated transcriptional activation of antioxidant genes [386]. Hypoxia-dependent activation of the cAMP/PKA signaling pathway plays an essential role in cell adaptation to hypoxia [387]. Changes to mitochondrial cAMP-PKA signaling affect the development of several physio-pathological conditions, such as neurodegenerative diseases [388]. Since 80% of ATP is produced in neuronal mitochondria, neurons are extremely sensitive to hypoxia. The Ccr4–Not complex is an effector of the Ras/cAMP pathway and negatively controls the transcription of stress-regulated promoters using two independent events; firstly, by altering the general distribution of TFIID and, secondly, by preventing activation of the zinc finger transcription factor Msn2 [51,159,341]. Msn2 is required for transcription of stress response element (STRE)-containing promoters, such as HSP12. HSP12 protein levels respond to stress and are increased by, for example, heat shock, oxidative stress, and DNA replication stress [389]. Directly, Not5, in association with Taf1, inhibits the loading of TBP to the TATA box at the HSP12 core promoter and indirectly the Ccr4 subunit negatively regulates HSP12 mRNA stability [51,390]. Conditions such as hypoxia and oncogenic mutations that activate the ERK1/2 signaling pathway can induce mitochondrial translocation of the cytosolic glycolytic enzyme phosphoglycerate kinase 1 (PGK1) [391]. As we discussed earlier (Section 9.3), the post-transcriptional repression of Dusp6 mRNA, the inhibitor of ERK1/2, is regulated in a Ccr4–Not-dependent mechanism. Mitochondrial PGK1 phosphorylates and activates PDHK1 to inhibit mitochondrial pyruvate metabolism and trigger the Warburg effect [392]. Reduction in mitochondrial pyruvate utilization, suppresses reactive oxygen species production, increases lactate production, and promotes brain tumorigenesis. Mutation in the *Saccharomyces cerevisiae* protein Caf1β/P0P2 (mammalian CNOT8) increases the mRNA level of the stress-regulated gene PGK1 5–10-fold compared to wild-type cells [393]. 

Tab182 (TNKS1BP1), a member of a larger mammalian, but not yeast, CNOT complex and contributes to radiation-induced DNA double-strand break repair through facilitating the interaction between DNA-PKcs and PARP1. The PARP1-mediated PARylation of DNA-PKcs leads to activation of DNA-PKcs auto-phosphorylation during DNA DSB repair [394]. Moreover, in the mammalian system Tab182 was identified in a screen of proteins which are highly phosphorylated at SQ/TQ sites by ATM or ATR in response to ionizing radiation-induced DNA damage [395]. Whether this represents a function of Tab182 independent of the rest of the CNOT complex has yet to be defined.

## 11. The CNOT Complex and Human Disease

As with any large multi-functional assembly of proteins mutations, deletions and aberrant protein expression have been linked to various human diseases-this is also the case for the CNOT complex [36]. In some cases, it has been possible to associate specific CNOT components to a clinical presentation whereas, in others, the malfunction of the whole complex can be seen as a causative/contributing factor.

As CNOT1 is essential for CNOT function it is not surprising that CNOT1 knock out mice die during development [36]. However, SNPs in the CNOT1 gene have been associated with susceptibility to B-cell pediatric ALL [396] and missense mutations have been frequently reported in colorectal, melanoma and uterine cancers (cBioportal & Gene online projects), (https://stuart.radboudumc.nl/metadome). Lesions in the CNOT1 gene have also been linked to other conditions. In recent detailed studies of patients with heterozygous missense, splice site and nonsense variants of CNOT1 it was found that they suffered from a range of neurodevelopmental phenotypes including intellectual disability, motor and speech delay, seizures, hypotonia, behavourial problems and holoprosencephaly [397,398,399,400]. Using a *Drosophila* model it was noted that the genetic interaction with autism-spectrum genes was impaired in the presence of mutated CNOT1 [400]. In addition, it has been reported that two unrelated individuals with semilobar holoprosencephaly (the incomplete separation of the forebrain during embryogenesis) have identical missense variants in the CNOT1 gene [398]. 

In a genome-wide association screen, 35 common variant myocardial repolarization interval loci have been identified that collectively account for ~8–10% of QT variations observed [401]. Some of the strongest QT-associating variants identified, centre around the CNOT1 gene. Using reporter assays it was shown that reduced CNOT1 expression contributes to abnormal QT-intervals [402]. Cardiac specific depletion of Not1 and Pop2 in *Drosophila* was either lethal or resulted in dilated cardiomyopathy, reduced contractility, or a propensity for arrhythmia [186,402]. Similarly, the loss of Not2, Not3, Not4 and twin affected cardiac chamber size and contractility [186,402]. The loss of NOT3 in heterozygous knock out mice resulted in impairment of cardiac contractility and susceptibility to heart failure although these could be reversed with HDAC inhibitors [186]. Significantly, it was observed that a common CNOT3 SNP in human patients correlated with altered cardiac QT intervals [186]. Similarly, variants in the CNOT1 gene, amongst others, has been associated with myocardial repolarization and Mendelian long QT syndromes [401]. A more recent study has shown that loss of CNOT1 or CNOT3 results in autophagy and cell death in mouse cardiomyocytes, long QT intervals and heart failure [340]. In hearts with depleted CNOT3 expression of the autophagy protein ATG7 was increased. Normally, CNOT3 binds to the poly (A) tail of ATG7 mRNA and regulates its length and ultimately level of protein expression [340].

Several other diverse diseases have been associated with mutations in the CNOT3 gene. A link has been established between CNOT3 and susceptibility to ankylosing spondylitis [403]. CNOT3 was identified as an oncogenic driver gene mutated in 7 out of 89 adult T-ALLs; its knockdown causes tumors in a sensitized *Drosophila melanogaster* model [404]. In a second study, CNOT3 loss-of-function mutations were found in ~7% of adult T5q-TALL cases [405]. Interestingly, in the same study down-regulation of CNOT6 was frequently observed [405]. 

PRPF31 is an RNA binding protein and splicing factor, which forms part of the spliceosome. Mutations in the gene cause retinitis pigmentosa, a condition marked by progressive retinal degeneration. The CNOT3 gene is located very close to PRPF31. It has been shown that expression of CNOT3 is inversely proportional to PRPF31 expression [195]. As CNOT3 binds to the PRPF31 promoter it was concluded that it acts as a negative regulator of PRPF31 and could be involved in the disease. 

The deadenylase subunits have also been linked to susceptibility to cancers of various kinds. CNOT6L was shown to have concordant expression patterns with tumour suppressor PTEN and display significant copy number loss in prostate cancer and glioblastoma [406]. Indeed, the depletion of CNOT6L transcripts results in a significant reduction in PTEN protein level, which negatively regulates the signal transduction PI3K-Akt pathway [406]. In clinical samples taken from ALL and AML patients CNOT6L and CNOT7 expression was down-regulated, while CNOT6 transcripts were slightly up-regulated [407]. A new functional susceptibility locus rs2453176 C > T of CNOT6 appears to be associated with lung cancer risk [408]. CNOT8 expression is elevated in a panel of colorectal carcinomas and associated metastases [409]. The expression is elevated in a panel of colorectal carcinomas and associated metastases [409]. 

Links have also been established between the Ccr4–Not complex and adipose and liver function. Targeted loss of CNOT3 in adipose tissue of knock out mice results in a decrease in white adipose tissue and inflammation. The mice also exhibit hyperinsulinemia, hyperglycemia, insulin resistance, and glucose intolerance coupled with difficulties in the maintenance of body temperature [410]. In other studies of mice with organ-specific knock out of CNOT3 loss of expression in pancreatic β-cells impairs glucose tolerance and leads to reduced β-cell mass and, eventually, diabetes [411]. This is consistent with the observation that CNOT3 is essential for murine β-cell maturation and that it is dysregulated in diabetic db/db mice [411]. 

Disruption of the complex in the liver leads to loss of metabolic homeostasis, at least partially through an increase in level of FG21, a major regulator of whole-body metabolism [412,413,414]. CNOT3+/− mice tend to be lean with adipose and liver tissues containing reduced lipid levels and with increased metabolism [412]. The expression of most mRNAs remains unchanged in these mice although there is an increase in mRNAs encoding energy metabolism related proteins [412]. A reduction in the level of metabolic enzymes has also been observed due to reduced pre-mRNAs in the absence of CNOT1, leading to hepatitis [414].

## 12. Concluding Remarks

The Ccr4–Not complex was originally recognised as an important regulator of mRNA deadenylation and gene expression in yeast. However, over the past decade it has become clear that it functions at numerous levels and is of central importance in mammalian cells as well. This review has set out to survey these multiple modes of action, gathering recently published insights and well-established material. We have considered recent advances in understanding the potential roles of the CNOT complex separately in cell cycle regulation, transcription-dependent replication stress, replication stress response, DNA damage repair, mRNA transport, and the control of steady state level of protein expression. For example, in 2001, Parker and colleagues hypothesized that certain mRNAs are marked for degradation in the nucleus during transcription. This suggestion was based on the premise that the CNOT complex could regulate both transcription and deadenylation. By 2018, Dronamraju and colleagues were able to confirm that the CNOT complex is recruited to the site of transcription in order to facilitate the degradation of a range of mRNAs, including mRNAs required for cell-cycle progression [202]. Similarly, considerable advances have been made, over the last two decades, in understanding the contribution of the Ccr4–Not complex to the regulation of the cell cycle and transcription. 

Our knowledge of the mode of action of the CNOT complex has been greatly enhanced by a number of structural and interaction studies, which have helped to demonstrate the contribution of each subunit, although the roles of some are still clearer than others. For example, it appears that the regulation of deadenylation and gene expression in yeast requires less components than are present in mammalian cells. Furthermore, two components, CNOT10 and CNOT11, absent from the yeast complex, stimulate deadenylation through stabilization of the RNA substrate. However, it seems likely that they may have other as yet unknown functions or, perhaps, provide additional subtlety of control of deadenylation required in metazoan cells. Future investigations should shed more light on the precise roles of CNOT10 and 11.

Over the past decade, interest in the mechanism of the formation and resolution of R-loops has increased markedly. There is considerable evidence to suggest a role for the Ccr4–Not complex in the process. We suggest that the complex functions at a number of points in the cell’s response to R-loops and it is hoped that this possibility will be clarified in the near future.

Although the constitution of the complex is fairly well understood in yeast and in humans it is possible that some of the well-defined binding partners might be considered Ccr4–Not complex components. For example, in fission yeast Mmi1 is very strongly associated with the complex. Mmi1 is essential for viability and represses the expression of transcripts required for entry into meiosis during normal vegetative growth; it seems, therefore, that the interaction between the complex and Mmi1 defines a function specific to fission yeast. It is possible that other binding proteins/possible components could also define specific functions. For example, Tab182 (TNKS1BP1) has been closely associated with the mammalian complex (it has been suggested to be denoted as CNOT12) although its functions are largely unresolved. All these points will have to be addressed before we have a full understanding of the CNOT complex.

Deletions and mutations in CNOT components have been associated with various clinical conditions. Because of the multiplicity of functions of the complex, it is not surprising that the loss of CNOT genes is lethal in embryonic mice. However, mutations in CNOT1 and CNOT3 have both been shown to be relatively common in certain ALLs and to be associated with cardiac functioning. Based on Kaplan–Meier plots, it has been observed that variations in the level of expression of CNOT components is associated with enhanced or reduced survival in different cancer types. For example, a high level of expression of CNOT7 is associated with appreciably enhanced survival in lung and gastric cancers, although it has little effect in breast cancer. On the other hand, low-level expression of CNOT3 is associated with enhanced survival in ovarian and gastric cancers. Exactly what role the complex plays in these cases is not yet understood.

## Figures and Tables

**Table 1:** A list of alternative names for equivalent CCR4–NOT complex subunits from *Saccharomyces cerevisiae* (baker’s yeast), *Homo sapiens* (human) and *Drosophila melanogaster* (fruit fly).

*Saccharomyces Cerevisiae*	*Homo Sapiens*	*Drosophila Melanogaster*	*Function*
NOT1/CDC39	CNOT1	NOT1	Scaffold
NOT2/CDC36	CNOT2	ReginaNOT2	Unknown but contributes to stabilization of the complex and RNA substrate recruitment
NOT3	Not present	Not present	Unknown but contributes to stabilization of the complex and RNA substrate recruitment
NOT5	CNOT3	NOT3	Interaction with ribosomes
NOT4/MOT2/SIG1	CNOT4	NOT4	Ubiquitin E3-ligase activity
CCR4	CNOT6, CNOT6L	Twin, CCR4	Deadenylase
CAF1	CNOT7, CNOT8	P0P2, CAF1	Deadenylase
CAF40	CNOT9 (RQCD1)	RCD1	Transcriptional cofactor
Not present	CNOT10	NOT10	Unknown but contributes to stabilization of the complex and RNA substrate recruitment
Not present	CNOT11 (C2orf29)	NOT11	Unknown but contributes to stabilization of the complex and RNA substrate recruitment
CAF130	Not present	Not present	Unknown
Not present	TNKS1BP1 (Tab182)	Not present	Multifunctional
